# An All-Organic Flexible Visible Light Communication System

**DOI:** 10.3390/s18093045

**Published:** 2018-09-12

**Authors:** César Vega-Colado, Belén Arredondo, Juan Carlos Torres, Eduardo López-Fraguas, Ricardo Vergaz, Diego Martín-Martín, Gonzalo del Pozo, Beatriz Romero, Palvi Apilo, Xabier Quintana, Morten A. Geday, Cristina de Dios, José Manuel Sánchez-Pena

**Affiliations:** 1 Electronic Technology Department, Universidad Carlos III de Madrid (GDAF-UC3M), Leganés, 28911 Madrid, Spain; jctzafra@ing.uc3m.es (J.C.T.); edlopezf@ing.uc3m.es (E.L.-F.); rvergaz@ing.uc3m.es (R.V.); cdios@ing.uc3m.es (C.d.D.); jmpena@ing.uc3m.es (J.M.S.-P.); 2 Electronic Technology Area, Universidad Rey Juan Carlos (DELFO-URJC), Móstoles, 28933 Madrid, Spain; belen.arredondo@urjc.es (B.A.); diego.martin.martin@urjc.es (D.M.-M.); gonzalo.delpozo@urjc.es (G.d.P.); beatriz.romero@urjc.es (B.R.); 3 VTT Technical Research Centre of Finland Ltd., 90571 Oulu, Finland; palvi.apilo@vtt.fi; 4 Bioengineering and Photonics Technology Department, Universidad Politécnica de Madrid (CEMDATIC-UPM), 28040 Madrid, Spain; x.quintana@upm.es (X.Q.); morten.geday@upm.es (M.A.G.)

**Keywords:** organic photodetector, organic light emitting diode, flexible electronics, visible light communication

## Abstract

Visible light communication systems can be used in a wide variety of applications, from driving to home automation. The use of wearables can increase the potential applications in indoor systems to send and receive specific and customized information. We have designed and developed a fully organic and flexible Visible Light Communication system using a flexible OLED, a flexible P3HT:PCBM-based organic photodiode (OPD) and flexible PCBs for the emitter and receiver conditioning circuits. We have fabricated and characterized the I-V curve, modulation response and impedance of the flexible OPD. As emitter we have used a commercial flexible organic luminaire with dimensions 99 × 99 × 0.88 mm, and we have characterized its modulation response. All the devices show frequency responses that allow operation over 40 kHz, thus enabling the transmission of high quality audio. Finally, we integrated the emitter and receiver components and its electronic drivers, to build an all-organic flexible VLC system capable of transmitting an audio file in real-time, as a proof of concept of the indoor capabilities of such a system.

## 1. Introduction

Visible light communication (VLC) which provides simultaneous illumination and wireless data transmission has received great attention in the past decade. VLC uses the visible spectrum from 380 nm to 750 nm corresponding to a frequency range from 430 THz to 790 THz, and it offers, in indoor environments, a real alternative to RF/microwave communication systems that have limited bandwidth and overcrowded spectrum. VLC presents several advantages over other data transmission technologies such as potentially high data rates [1,2,3], hundreds of THz of license-free channels [4], safety of the data transport medium compared to RF system in medical environments [5], and low power consumption. Potential applications of VLC systems include communications in healthcare environments [6,7,8], indoor positioning systems [9], smart lightning [10], automotive and aviation applications [11] and underwater communications [12].

Moreover, there has been a growing interest in the integration of organic optoelectronic components in VLC systems since they offer advantages over traditional silicon based devices, such as low cost and low environmental impact production, lightweight, large-area processing and mechanical flexibility [13,14,15,16,17]. In this context, organic light emitting diodes (OLEDs) and organic photodetector (OPDs) must meet a list of requirements in order to be integrated in commercial products. In recent years, OPDs have attracted great attention and, to date, these devices report competitive figures of merit; high responsivity up to ~0.4 A/W [18], high external quantum efficiency of 65–70% [19,20], high detectivity of 3.21 × 10^13^ Jones and low dark current density of 0.31 nA cm^−2^ [19].

One of the most straightforward advantages of integrating organic optoelectronic components in VLC is the possibility of making the whole system flexible: emitter, receptor and electronic drivers. This will open the possibility of integrating these systems in wearable clothes and industrial fabrics, as reviewed by Nathan et al. [21]. Flexible electronics may be the basis of promising applications in various fields such as healthcare, displays, energy conversion, and communication and wireless networks. Flexible organic light emitting diodes (FOLEDs) and flexible OPDs present advantages such as thinness, sturdiness, conformability, and the possibility of being rolled when not being used [22,23,24]. The expansion of flexible organic devices is associated to advances in materials; e.g., conductive polymers, thin films, printable metals [25] and low-cost production techniques such as roll-to-roll or ink-jet printing [26]. Nevertheless, the performance and lifetime of flexible devices is affected by degradation of the optical, electrical, and mechanical properties of the device substrates and organic layers [27,28,29,30].

So far, several efforts have been made to achieve an all-organic VLC system, using the work of Haigh et al. as starting point [31]. Later works soon overcome the record speed and other features [32,33,34]. Other works have demonstrated the performance of OLEDs in VLC systems, focusing on modulation or on the radiation pattern [35,36], and achieving speed values over 50 Mb/s. Interestingly, recent studies have shown their good performance in VLC channels even while being bent [37].

In this work, we show a proof of concept of an all-organic flexible VLC prototype system used in a real application. We use both flexible organic emitter and detector, and the system drivers are mounted also on flexible printed circuit boards (PCBs). The OPD is taking advantage of a flexible solar cell structure fabricated using the Roll2Roll technique, which could be used eventually both as energy harvesting and photodetector in wearable applications. The system has been successfully demonstrated indoors, transmitting and receiving an audio signal with enough quality to be reproduced on a standard audio system. The presented system has a straightforward application in wearable technologies.

## 2. Materials and Methods

### 2.1. Flexible OPD Fabrication and Characterization

**Figure 1:**
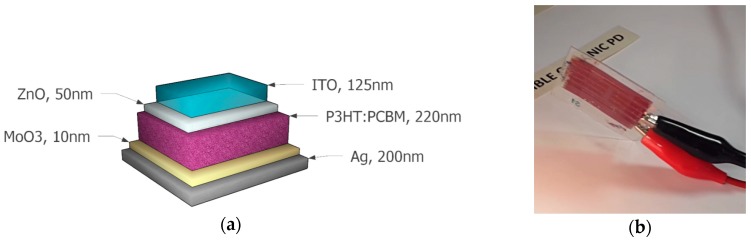
(**a**) OPD layer structure. The device area is 25 mm^2^; (**b**) OPD in use.

The device structure of the laboratory printed OPD devices is shown in Figure 1a. Please note that the primary function of this device structure is to act as an organic solar cell, but in this system it is used as an organic photodetector. The OPD devices were constructed on top of ITO-coated PET roll (40–60 Ω sq^−1^) purchased from Eastman (Kingsport, TN, USA). The ITO was patterned with Isishape HiperEtch 09S Type 40 paste (Merck KGaA, Darmstadt, Germany) as a negative image to the desired pattern. Roll to roll (R2R) rotary screen printing was performed with a printing speed of 1.5 m min^−1^. After printing, the printed film was placed directly into the R2R hot air drying units set to a temperature of 140 °C for 160 s. The paste was washed off in baths of water and 2-propanol. After patterning, the surface was ultrasonically washed and dried in the R2R process. ZnO nanoparticle suspension (Avantama, Stäfa, Switzerland) was used for the electron transport layer. The ZnO ink was kept in an ultrasonic bath for 10 min prior to the printing. The ZnO layer was gravure-printed in the laboratory (Schläfli, Zofingen, Switzerland) and dried at 120 °C for 5 min in an oven. The printing plate contained engravings with a line density of 120 lines cm−1. Regioregular P3HT (#4002-E, Rieke Metals, Lincol, NE, USA) was used as the donor and [60]PCBM as acceptor (purity 99.5%, Nano-C). The photoactive layer of P3HT:[60]PCBM was gravure printed in laboratory with a printing plate having a line density of 120 lines cm^−1^. The photoactive layer was slowly dried, and devices were transferred to the glove box for pre-thermal annealing (120 °C for 10 min) and thermal evaporation of MoO_3_ and Ag. Subsequently, the hole transport layer of MoO_3_ (10 nm) and hole contact of Ag (200 nm) were thermally evaporated. Devices were encapsulated with flexible barrier substrate and pressure sensitized adhesive.

Current-voltage (I-V) curves were measured with a FAS-2 Gamry four-probe potentiostat-galvanostat, a green laser and a lens set-up to ensure that the light fell upon the OPD surface homogeneously. EQE was recorded using a lock-in amplifier, a GaSb-detector, a halogen tungsten lamp and a mechanical chopper. The OPD frequency response was measured modulating the optical emission with a transconductance amplifier that varied the current flowing through the LED. The OPD was reverse biased and connected to a transimpedance amplifier with an equivalent input impedance of 50 Ω. Frequency response was recorded using a high sensitive lock-in amplifier (Zurich Instruments HF2LI, Zurich, Switzerland), suitable for frequencies from DC up to 50 MHz.

### 2.2. VLC System Layout

**Figure 2:**
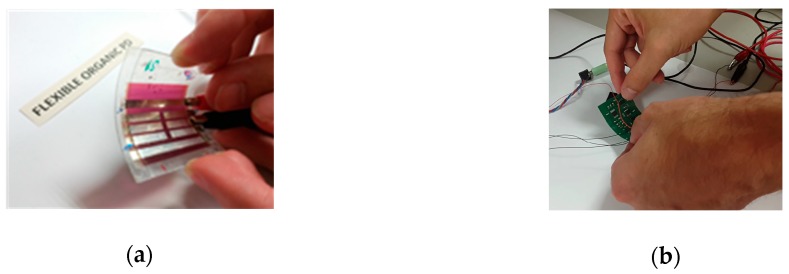
(**a**) Flexible OPD; (**b**) flexible electronic driver.

We have validated the performance of the flexible device in a real and novel application by designing and developing a full-flexible organic VLC system. The aim is to show the possibilities of the system in a real environment in which the requirements of bandwidth, responsivity and flexibility are mandatory. The receptor system is intended to be integrated in wearables, since both the photodetector (Figure 2a) and the electronic driver (Figure 2b) are built on flexible substrates.

In this way, a communication link is established between a source of light, typically an OLED lamp emitting under modulating conditions, and a gadget that any user could wear integrated on a cap, jacket, trousers or even the skin. Hospitals, museums, crowded buildings or big malls are examples of possible scenarios where this system is ideal to overcome the drawbacks of low or collapsed WiFi access, or to ensure privacy in the communication by using a punctual light source that could be driven using a home automation system.

**Figure 3:**
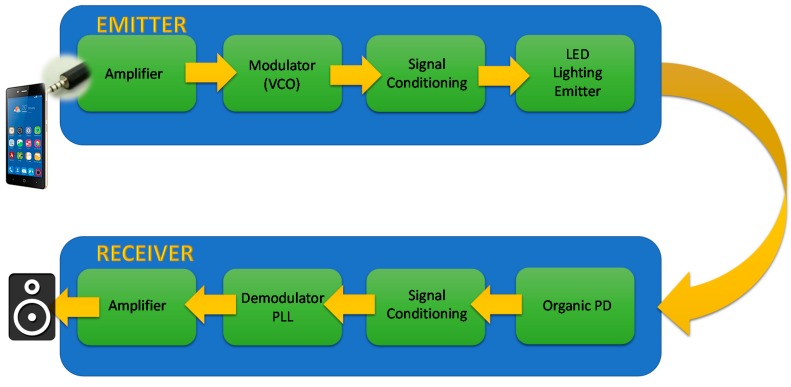
The VLC system block diagram.

**Figure 4:**
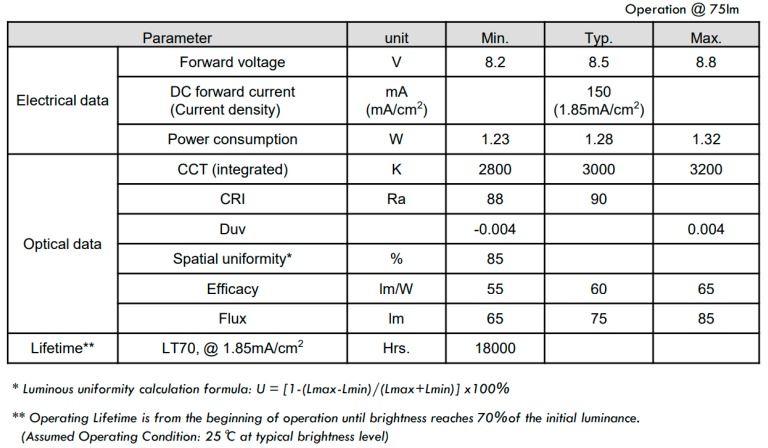
OLED electrical and optical characterictics [38].

The basic scheme is shown in Figure 3. A cell phone is the data transmission source, in this case an audio file, though the data source could come from any device provided that the bandwidth is sufficient as to support the data link. In a real application, the emitter system would be integrated in the luminaries of the roofs or walls in a building, or in a desk lamp. This emitter driver is composed of a circuit for transmitting the data fully made of off-the-shelf components, such as, a voltage amplifier as conditioning circuit for the input signal, an FM modulator based on a voltage controlled oscillator (VCO), and a signal conditioning circuit to modulate the current of the power transistors driving the OLED panel. This circuit follows the indications of the integrated circuit CD4046 datasheet. The last one is a 99 × 99 × 0.88 mm LG N6SA30 commercial panel with a power consumption of 1.28 W and an efficacy of 60 lm/W, which could be used as a spotlight in any of the abovementioned applications. The electrical characteristics and optical data are shown in Figure 4.

**Figure 5:**
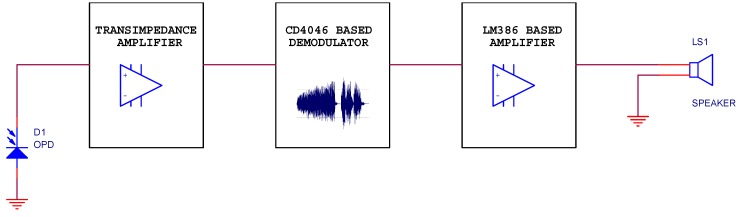
The low-cost VLC receiver circuit block diagram.

The receiver circuit (block diagram in Figure 5, schematics in Supplementary Figure S1) contains the fabricated flexible OPD as the light-to-current transducer, and a conditioning circuit that receives the signal from the OPD and converts it into voltage using a simple transimpedance amplifier. The information received is demodulated in frequency with a phase locked loop (PLL) based on a CD4046 integrated circuit. This commercial circuit was also used as FM modulator in the emitter circuit, but configured to play the VCO role. Finally, an amplifier circuit using an LM386 operational amplifier drives a loudspeaker.

The bandwidth requirements of this simple system are determined by those of the audio system, i.e., at least around 44 kHz to properly transmit the signal, according to the Nyquist criterion. The system design must take into account that any of the electronic components used may limit the bandwidth, therefore the authors payed special attention to the low pass filters using ICs CD4046 and LM386, the first one to retrieve the modulated signal and avoid aliasing, and the second one to generate a clean audio signal to drive the speaker.

## 3. Results and Discussion

In this section we show the characterization of the main parts of the VLC system, i.e., the OPD, the OLED and the performance of the whole system. The goal is to show that our whole-flexible setup is able to maintain an audio link in an indoor environment.

### 3.1. OPD Optolectronic Characterization

**Figure 6:**
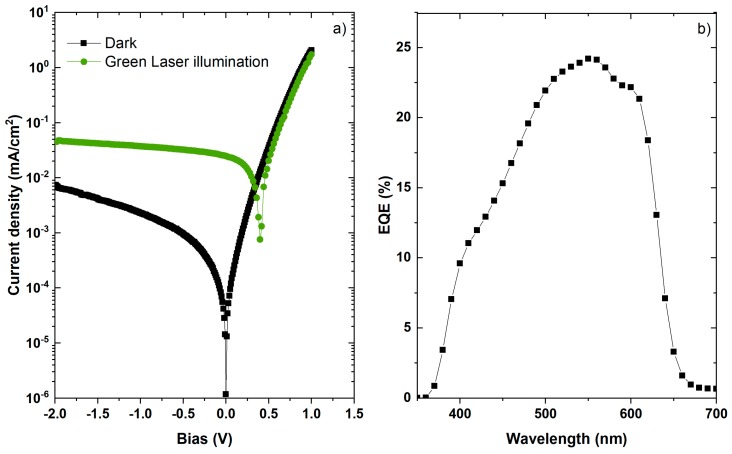
(**a**) J-V characteristic in dark and under green-laser illumination (532 nm); (**b**) EQE at zero bias.

Figure 6a shows the current-density voltage (J-V) characteristic of the flexible OPD in dark and under green-light illumination. The dark curve exhibits a shunt resistance (R_SH_) of around 2 MΩ, reverse current of 1.42 × 10^−5^ mA/cm^2^ at 0 V, and R_s_ = 40 Ω at 1 V that may be attributed to poor electrical contacts at the ITO-covered flexible substrates. The photocurrent density under green-light illumination varies from 4.7 × 10^−2^ mA/cm^2^ to 2.5 × 10^−2^ mA/cm^2^ between 0 V and −2 V for a 0.5 mW incident light power. The calculated responsivity, R (A/W) results in 0.144 A/W at 532 nm and 0.065 A/W at 635 nm, which agrees well with the EQE at zero bias shown in Figure 6b exhibiting a maximum of 24.3% at 550 nm. Using the responsivity value, the current value of 2 × 10^−3^ mA/cm^2^ at −1 V (Figure 6a), and following the methods described in [33], we have calculated a noise equivalent power (NEP) of 2.78 × 10^−12^ W/Hz^−1/2^, and a specific detectivity of D* = 1.8 × 10^11^ Jones. The linearity is also over 99% (see the Supplementary Materials).

The frequency response was measured using the lock-in amplifier, sweeping the frequency from 10 Hz to 10 MHz. The bandwidth of the OPD is obtained by illuminating the sample with a pulsed red LED biased with a square signal driven by the lock-in amplifier. The response of the OPD was conditioned by a transimpedance amplifier, with bandwidth over 10 MHz to avoid affecting the measured response. Using this amplifier, the OPD was reverse biased between 0 and 2 volts.

**Figure 7:**
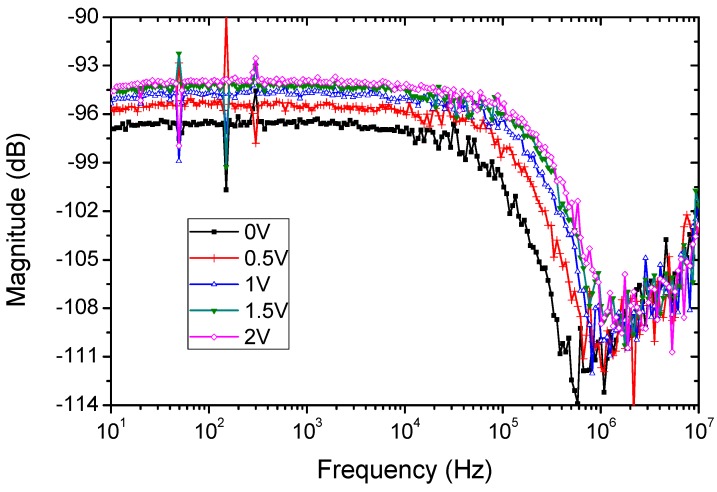
OPD bandwidth measured using a lock-in amplifier.

The bandwidth increases from 100 kHz for the OPD biased at 0 V, up to 200 kHz for the OPD reverse biased at 2 V (see Figure 7). As expected, reverse biasing improves the bandwidth due to the increase of carriers. In any case, for the audio file Li-Fi transmission purpose, the OPD widely covers the bandwidth requirements to be used as optical receptor.

**Figure 8:**
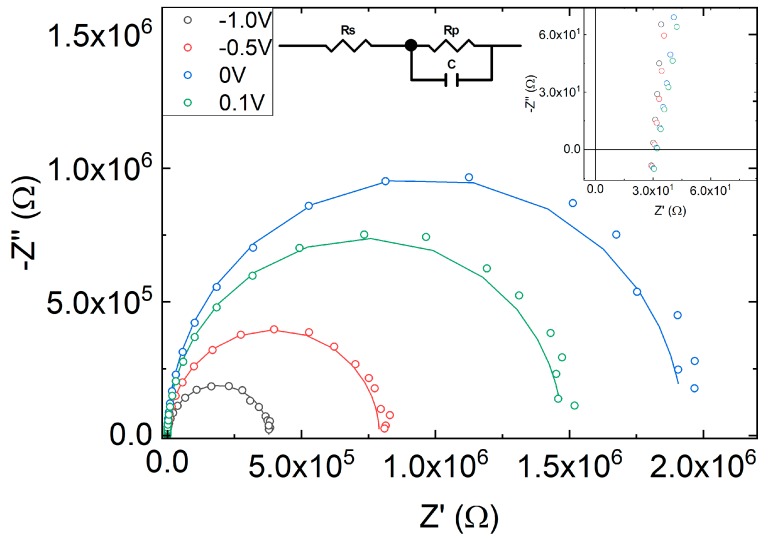
Cole-Cole diagram in dark at different bias (symbols). Solid lines show the fit to the circuit in the inset. The upper right inset shows the high frequency part (the bottom-left of Cole-Cole plot).

In addition, impedance spectroscopy has been measured in the 1 Hz–3 MHz frequency range at different voltages in dark conditions. Figure 8 shows the electrical impedance at 0.1, 0, −0.5 and −1 V. The spectra resemble a semicircle that has been modelled with an R_p_C element in series with resistance, R_s_. R_p_ accounts for the parallel of the shunt resistance (modelling leakage current due to fabrication defects) and the diode dynamical resistance. C is the total capacitance of the device, which, in general, is a combination of geometrical and chemical capacitance. At reverse bias C is dominated by the geometrical capacitance. R_s_ accounts for the series resistance modelling metallic contacts and wires.

Circuital parameters are obtained from the fit of the impedance spectra in dark conditions to the circuit of the inset. R_s_ has a quasi-constant value of 30 Ω, as can be obtained in the high frequency part of the Cole-Cole plot (Figure 8, inset). C ranges between 6 and 9 nF, and R_p_ ranges from 0.37 to 1.9 MΩ in the voltage range from −1 V up to 0.1 V. A simple calculation using the 25 mm^2^ area, a relative dielectric permittivity of around 4 and an active area thickness of 230 nm results in a capacitance of the order of 4 nF, close to the one measured, reinforcing that the geometrical capacitance is the main contribution to the overall capacitance.

According to Ref. [33] the device bandwidth can be estimated from the values of the diode capacitance. The capacitance obtained from the fit of impedance of the OPD under green LED is 12.6 nF at 0 V. This value is close to the one obtained without illumination, thus showing again that the capacitance is mainly the geometrical one. This value yields a cut-off frequency of approximately 250 kHz, which is in the same order of the measured value shown in Figure 7.

### 3.2. OLED Characterization

As the luminaire of the VLC system, we use a commercial flexible OLED (N6SA30C) from LG Chem© (Seoul, South Korea) with 3000 K of correlated color temperature, operating temperature between 0 to 40 °C, 8.1 V forward voltage and luminous flux of 75 lm. The manufacturer claims r 40,000 operation hours and 1.8 W of power consumption. It is a thin light weight square luminaire of 20 g and dimensions 99 × 99 × 0.88 mm.

**Figure 9:**
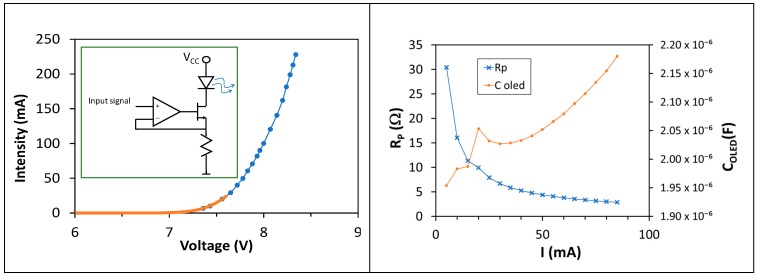
(**Left**) I-V curve measured with two methods: Gamry potentiostat (orange dots) and customized circuit (blue dots, inset). (**Right**) Circuital parameters of the equivalent circuit of the OLED (inset) versus the applied current obtained using impedance spectroscopy.

We measured the I-V curve using the Gamry potentiostat, though it only reached up to 7.6 V, which is under the standard operation for this LED (Figure 9 Left). In order to increase the operation bias, we built a classical current source using an operational amplifier with a MOSFET IRF530 circuit in the negative feedback loop (Figure 9 Left, inset). The OLED current is established with the voltage input signal applied to the op amp non-inverting terminal. By using our current source circuit we obtained the same curve as with the Gamry, but increasing bias up to 8.3 V. The current measured at that point is around 228 mA. It is clear that the forward voltage is around 8 V, as expected.

We characterized the OLED also using impedance spectroscopy (Figure 9 Right). The Cole-Cole diagram was fitted to an equivalent circuit composed of a resistance R_S_ in series with the parallel of a shunt resistance (the dynamic resistance of the diode, R_P_) and a capacitor C_OLED_, a similar circuit to that of the OPD (Figure 8 Right, inset). Due to the limitations of our model 1260 impedance analyzer (Solartron, West Sussex, UK), we injected only a current range from 5 to 85 mA. Fits reveal that the OLED has a C_OLED_ ranging from 1.95 µF at 5 mA up to 2.34 µF at 85 mA, with a constant R_S_ of 2.41 ± 0.02 Ω. Dynamic resistance R_P_ ranges from 30.4 down to 2.9 Ω when increasing the operation current. All these values have an error under 2%.

**Figure 10:**
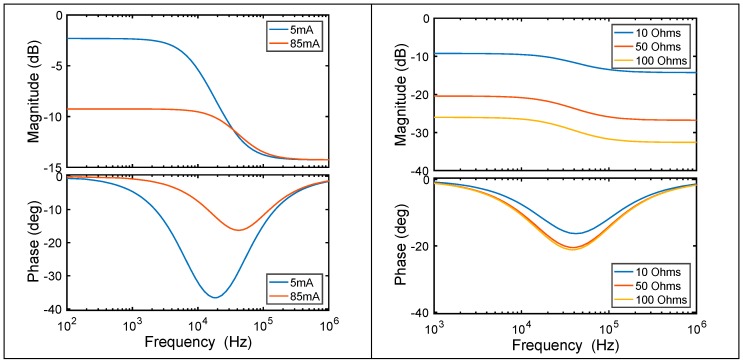
Bode plots of the transfer function of the emitter driver and OLED. (**Left**) Variying the OLED bias current, 5 mA and 85 mA (output impedance of the driver circuit is R_O_ = 10 Ω). (**Right**) Variying R_O_ (10, 50 and 100 Ω) with OLED bias current 85 mA.

The cutoff frequency is given by f_c_ = (2π × C_OLED_ × (R_S_ || R_P_))^−1^ according to its equivalent circuit (Figure 9 Right, inset). Biasing the OLED with 5 mA, we obtain a cutoff frequency of 9.22 kHz. Increasing the bias current up to 85 mA the cutoff frequency increases up to 56 kHz (Figure 10 Left), though this could be even improved by increasing the bias current, as R_P_ would decrease (Figure 9 Right). Moreover, increasing the output resistance of the driving circuit, R_O_, reduces the signal at the OLED (Figure 10 Right), worsening the OLED performance. As a conclusion, the OLED will not limit the bandwidth in an audio link, providing it is polarized at a high enough bias current and the driving circuit is carefully selected.

### 3.3. VLC System

**Figure 11:**
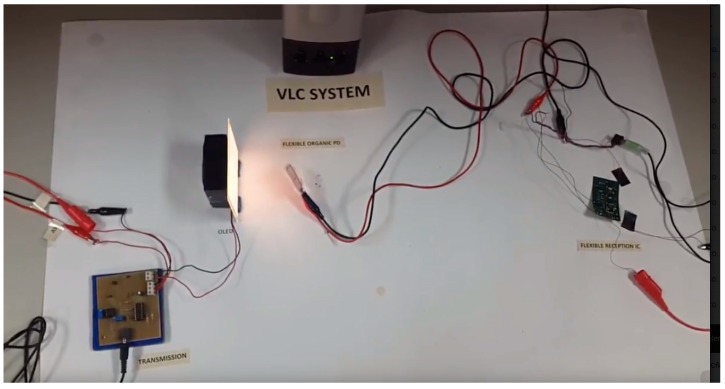
View of the proof of concept VLC built in lab.

A demonstration video of the proposed proof of concept can be found in the Supplementary Materials of this paper. A snapshot of the video is shown in Figure 11 and serves as a view of the entire system. The full-organic flexible VLC system was built in the lab as described in Section 2.2. On the left side of Figure 11, the transmission circuit is receiving the signal coming from the jack connection of a cell phone. The information is FM modulated, i.e., the signal with varying frequency is superimposed to the OLED DC bias current needed for illumination, which produces an imperceptible modulation in the light. The light is received at the OPD, and then conditioned with a flexible driver (see right side of Figure 11) as described in Figure 4. The receptor circuit sends the demodulated and processed signal to the loudspeaker. The signal is reproduced at high quality, with the only limitations coming from the output circuit and the loudspeaker rather than the OLED and the OPD.

The limitations of the system are mainly coming from the electronics and OLED rather than the OPD. This can be due to the use of the simplest design and electronic components, both in emission and reception circuits. Transmission is achieved using a simple modulating integrated circuit, by means of a voltage controlled oscillator tuned in the carrier frequency by the connection of external passive components: resistances and capacitors. The reception circuit is demodulating the signal using the same commercial integrated circuit but configured in a phase locked loop, also tuned using external resistances and capacitors. The use of simple and cheap both OLED luminaire and integrated circuits finally could limit the frequency response of the system. Nevertheless, it can operate at a frequency high enough to be used in audio applications. This should be at least 44 kHz, and our system begins to decay at 40 kHz. According to the Nyquist criterion, we may lose information at frequencies over 20 kHz when retrieving the audio signal, but those frequencies are over the usual audio frequency threshold for humans. The key point for the proper operation system is to bias the OLED near the forward voltage, which eases the switching to transmit the information without affecting the lighting operation, and enhances the switching speed of the OLED.

The performance of the audio system is not significantly affected by the bending of the OPD, as the video shows (nevertheless, further research on the performance under bending must be made in future works). Then, this system can be used in a wide variety of applications. We propose one here: as the OLED is suitable for ambient illumination, it could send customized and personal information to a specific target. In a smart automated building, for instance, it could be suitable to transmit information via audio files to a person that wears an audio system like this one (receptor + headphones instead of a loudspeaker) integrated in a wearable. Using the flexible condition of both the OPD and the circuit, the reception system can be integrated in a cap or clothes, or even in a small hearing-aid. In an office building this could be used to send information to a specific worker. It should be very useful to help workers suffering from any kind of disability or limited hand use. The transmitted information helps the worker to do the job, giving instructions while operating. A bidirectional system can add a handbook interactive query, a usual environment for astronauts at the International Space Station. Another interesting environment could be the illumination of a piece of art in a museum, where the information can be transmitted to the visitor placed in front of it, using a passive reception system in a hearing device. The visitor using this system will not be aware of the fact that the lighting is transmitting the information played in the headphone, since the light environment is indistinguishable of the OLED for the human eye.

## 4. Conclusions

We have demonstrated a fully organic flexible visible light communication system using off-the-shelf components, flexible circuits, a flexible commercial OLED and a flexible organic photodiode manufactured in a roll-to-roll process. This demonstration follows an integral methodology: first, we designed the whole system, setting the objective of transmitting an audio file from an OLED luminaire to a flexible OPD. Second, we performed a full characterization of every component of the link in terms of frequency response to guarantee the require data transmission rate. Third, we built the complete system, connecting a mobile phone to our emitter circuit, modulating the OLED transmission, sending the light signal to a flexible OPD, and transducing the signal with a demodulator flexible circuit that finally sends the audio signal to a loudspeaker. Both the emitter and receiver drivers are made as simple as possible, using the minimum number of commercial components, demonstrating a low cost VLC proof of concept. The only feature that increases the price of the system is the flexible condition of the printed circuit board.

Potential system improvements are related to enhance the system bandwidth. This requires increasing the frequency response of the electronics and emitter/receiver components. Large area commercial OLEDs are the bottleneck of the system. The bigger the OLED, the longer the operation distance but the slower is the switching. The future goal is to design the driving circuits operating over hundreds of MHz, both in emission and transmission, and to build an OPD that could support this operation frequency. Such bandwidths would even allow video transmission.

## Supplementary Materials

The following are available online at http://www.mdpi.com/1424-8220/18/9/3045/s1. Video S1: Demonstrator video of the flexible organic VLC system using an audio signal. Figure S1: Schematics of the reception circuit. Figure S2: Linear response of the OPD, and a linear fit with its correlation *R^2^* coefficient.
