# Supplementary material for: An All-Organic Flexible Visible Light Communication System
Source: Sensors (Basel). 2018 Sep 12;18(9):3045. doi: 10.3390/s18093045 (PMC6165323; doi:10.3390/s18093045)
Supplement: Supplementary file 1 [file sensors-18-03045-s001.zip › sensors-341564.pdf]

Supplementary information to:

All-organic flexible visible light communication system

César Vega-Colado[1]*, Belén Arredondo[2], Juan Carlos Torres[1], Eduardo López-Fraguas[1], Ricardo Vergaz[1], Diego Martín-Martín[2], Gonzalo del Pozo[2], Beatriz Romero[2], Palvi Apilo[3], Xabier Quintana[4], Morten A. Geday[4], Cristina de Dios[5], and José Manuel Sánchez-Pena[1]

[1] GDAF-UC3M, Dep. Tecnología Electrónica, Universidad Carlos III de Madrid
[2] DELFO-URJC, Dep. Tecnología Electrónica, Universidad Rey Juan Carlos, Madrid
[3] VTT Technical Research Centre of Finland Ltd., Oulu 90571, Finland
[4] CEMDATIC-UPM, Universidad Politécnica de Madrid, Madrid
[5] LuzWavelabs Inc. and Dep. Tecnología Electrónica, Universidad Carlos III de Madrid

1. Complete circuit diagram of the system.

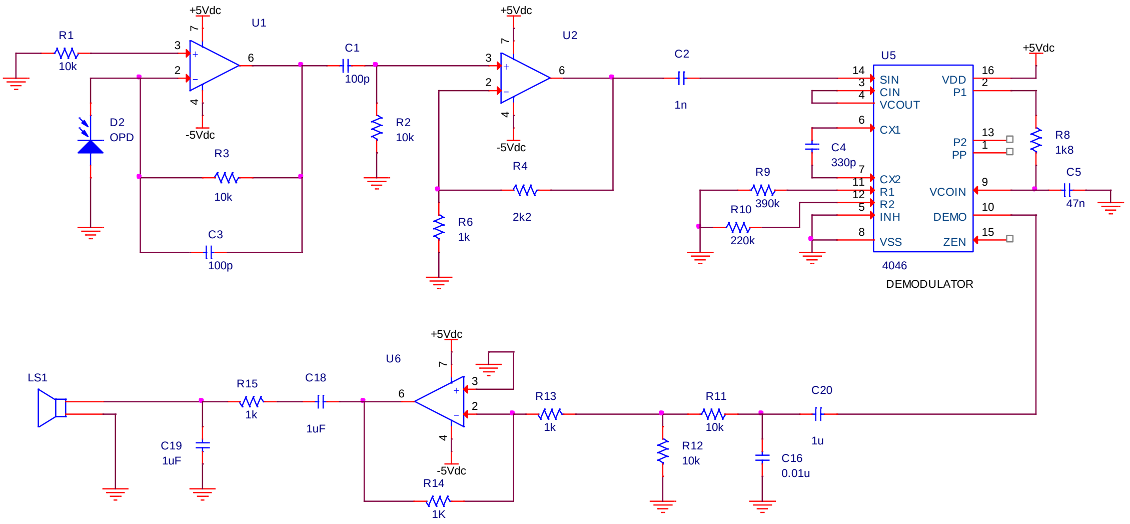

Fig. S1. Schematics of the reception circuit.

2. Detectivity computation:

The computation follows the reference:

Arredondo, B.; Dios, C. de; Vergaz, R.; Pozo, G. del; Romero, B. High-Bandwidth Organic Photodetector Analyzed by Impedance Spectroscopy. IEEE Photonics Technology Letters 2012, 24, 1868–1871, doi:10.1109/LPT.2012.2217488.

We have a responsivity of R = 0.144 A/W.
Under dark conditions, at -1V applied, we get $2 \cdot 10^{-3}$ mA/cm^2.
As our device has an A= 0.25 cm^2 area, we get a dark current of I_D = 0.5 μA in this case.
Then the NEP (Noise Equivalent Power) is:

$$NEP = \frac{\sqrt{\langle I_N^2 \rangle}}{R} = \frac{\sqrt{2eI_D}}{R}$$

with *e* the charge of the electron, giving $2.78 \cdot 10^{-12}$ W/Hz$^{-1/2}$.
Then, the detectivity can be obtained as:

$$D^* = \frac{\sqrt{A}}{NEP}$$

Leading to the $1.8 \cdot 10^{11}$ Jones value that we have remarked in the text.

3. Linear response

Regarding the linear response of the OPD, we have measured it using a setup based on a LED set with a narrowband emission centred at 593 nm and a simple transimpedance amplifier. We show here the response, in a plot of generated current vs received optical power. The correlation coefficient to a linear fit is shown in the graph, and its value, 0.996, reveals a remarkable linearity.

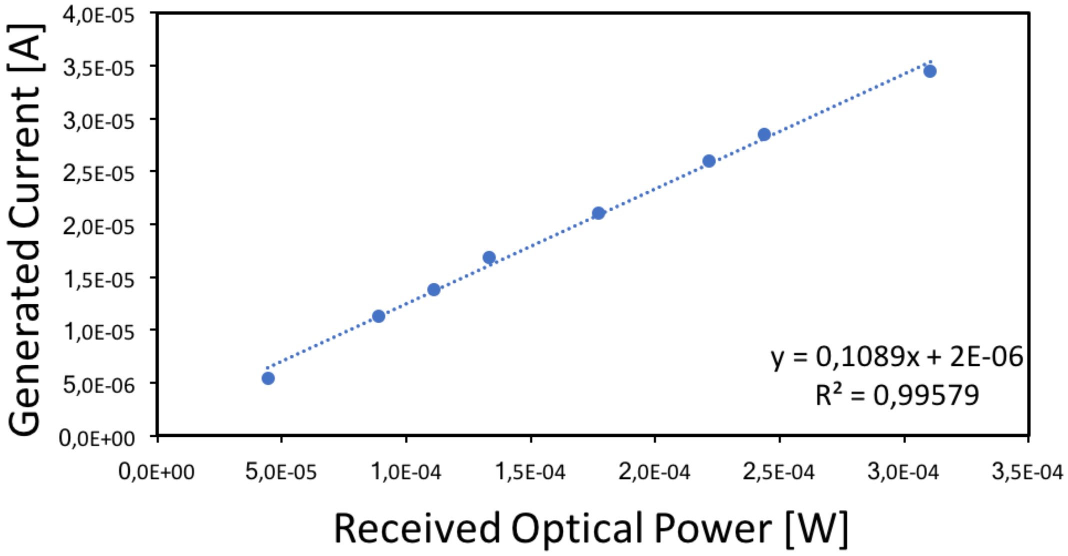

Fig. S2. Linear response of the OPD, and a linear fit with its correlation R^2 coefficient.
